# Empowering Stories: Transportation into Narratives with Strong Protagonists Increases Self-Related Control Beliefs

**DOI:** 10.1080/0163853X.2018.1526032

**Published:** 2018-10-05

**Authors:** Maj-Britt Isberner, Tobias Richter, Constanze Schreiner, Yanina Eisenbach, Christin Sommer, Markus Appel

**Affiliations:** a Department of Psychology, University of Kassel, Kassel, Germany; b Department of Psychology IV, University of Würzburg, Würzburg, Germany; c Human Computer Media Institute, University of Würzburg, Würzburg, Germany

## Abstract

Several studies have shown that narratives can influence readers’ beliefs about themselves. In the present study, our goal was to investigate whether stories portraying a strong protagonist can positively influence recipients’ beliefs of being in control of events in their own lives (self-related control beliefs). Experiment 1 showed that narratives in both written text and video form with protagonists displaying high versus low self-efficacy can, at least temporarily, affect recipients’ own self-related control beliefs when they experience strong transportation into the stories. In addition, the results suggest that recipients’ perceived ability to generate vivid mental imagery affects their transportation into and identification with characters in texts versus films. Experiment 2 manipulated transportation and identification experimentally and showed that the effect of this manipulation on self-related control beliefs was indeed mediated by experienced transportation and identification. The results are discussed within the framework of the Transportation Imagery Model of narrative persuasion.

## Introduction

“Fairy tales are more than true: not because they tell us that dragons exist, but because they tell us that dragons can be beaten.” —Neil Gaiman

This paraphrase of a passage from G. K. Chesterton’s *Tremendous Trifles*, which fiction author Neil Gaiman used as an epigraph to his book *Coraline*, quite powerfully describes the impact that fairy-tales (and other kinds of stories) can have on people. Many stories—intentionally or unintentionally, explicitly or implicitly—convey ideas and messages that can influence story recipients’ own views and beliefs about the world. A growing body of research attests to this persuasive potential of narratives, which is particularly striking as many stories, unlike other forms of persuasive communication, make no claim regarding the truth or real-life accuracy of what they portray. The Transportation Imagery Model proposed by Green and Brock (2002) attributes this persuasive potential of narratives to their ability to evoke vivid mental imagery and transport the recipient into a different world (*transportation*, Gerrig, 1993). In the present study, we tested these theoretical assumptions by investigating whether narrative impact is moderated by self-reported transportation into a persuasive story (Experiment 1) and whether an experimental manipulation of transportation indeed affects the persuasive outcome (Experiment 2), as predicted by the model.

As the target for persuasion, we chose a theme which, in line with the opening quote by Neil Gaiman, underlies many popular narratives—namely, beliefs regarding the malleability of one’s own fate. Our assumption is that stories featuring strong protagonists who display high self-efficacy by overcoming challenges and adversities should, at least temporarily, also increase recipients’ own self-related control beliefs provided that recipients are highly transported into the respective stories. Finally, while a moderating effect of transportation is in line with the Transportation Imagery Model, a stronger test of the model would be to show that a successful experimental manipulation of transportation also affects the persuasive outcome and that this effect is mediated by recipients’ self-reported transportation. In testing these hypotheses, we also explore the role of identification with protagonists as another potential moderator and mediator of narrative impact and the influence of the perceived ability to generate vivid mental imagery on transportation and identification in different media (texts vs. films).

### Narrative persuasion and the transportation imagery model

Stories often convey messages about the world that can influence recipients’ own views and beliefs. This can be done explicitly (as in the “moral of the story” in fables), but it can also be implicitly conveyed—for example, via the behavior of the characters and its consequences. Empirical evidence shows that narratives can be more persuasive than non-narrative formats (e.g., Djikic, Oatley, Zoeterman, & Peterson, 2009; Murphy, Frank, Chatterjee, & Baezconde‐Garbanati, 2013) and that narrative persuasion follows different principles and involves different mechanisms than rhetorical persuasion (e.g., Green & Brock, 2002; Slater & Rouner, 2002). This led Green and Brock (2002) to devise a model specifically tailored to narrative persuasion, the *Transportation Imagery Model*, which comprises five postulates and attributes the persuasive potential of narratives to their ability to induce a state called transportation. The term transportation was first introduced to the field by Gerrig (1993). It is based on the notion that the recipient of a narrative, in a metaphorical sense, travels to a different world, inducing a holistic experiential state marked by changes in cognitive, emotional, and attentional processing, as well as by mental imagery. Green and Brock (2000) developed a retrospective self-report scale that captures transportation in all of these facets, of which Appel, Gnambs, Richter, and Green (2015) devised a short form with comparable reliability despite a reduced number of items. These scales are, to date, the only reliable tools to comprehensively measure the construct of transportation, although means of measuring aspects of transportation concurrently and objectively are currently being explored (e.g., Bezdek & Gerrig, 2017; Hartung, Burke, Hagoort, & Willems, 2016; Sukalla, Bilandzic, Bolls, & Busselle, 2016).

### Evidence for the transportation imagery model

In line with the postulates of the Transportation Imagery Model, previous studies have found transportation to be a significant moderator of narrative impact (e.g., Richter, Appel, & Calio, 2014; Sestir & Green, 2010). While this finding is in line with the idea that transportation plays a causal role in narrative persuasion, much stronger evidence for this is the finding that an experimental manipulation of transportation directly affects the persuasive impact of a narrative and that this effect is mediated by recipients’ self-reported transportation. Empirical evidence of this kind is still sparse (for exceptions, see Green & Brock, 2000, and Escalas, 2004), and a number of studies have failed to find evidence for a mediating role of transportation in narrative persuasion—either because the experimental manipulation of transportation failed (e.g., Green & Brock, 2000, experiments 1–3), or because it did not have a corresponding effect on the persuasive outcome (e.g., De Graaf, Hoeken, Sanders, & Beentjes, 2009; Zwarun & Hall, 2012). Other studies reporting successful manipulations of transportation either did not test for mediation (e.g., Sestir & Green, 2010), were not concerned with persuasion (e.g., Shedlosky-Shoemaker, Costabile, DeLuca, & Arkin, 2011), or were interested in the persuasive effects of ads which interrupt high versus low transportation narratives, not in the persuasive effects of the narrative itself (Wang & Calder, 2006). Therefore, further evidence for a mediating role of transportation in narrative persuasion that relies on a successful experimental manipulation of transportation seems desirable.

One major challenge to testing this mediation is finding an effective means of manipulating transportation. Many attempts have been made at this, with overall quite inconsistent results (for a meta-analysis, see Tukachinsky, 2014). In summary, there seems to be as of yet no method that reliably works to manipulate transportation while keeping conditions comparable in all other respects. However, one recent method that seems promising in this regard is the use of (positive vs. negative) reviews presented before the narrative. This is not only an elegant method as it does not require any changes to the narrative itself, but it has also been used successfully to manipulate transportation in multiple experiments (Gebbers, De Wit, & Appel, 2017; Shedlosky-Shoemaker et al., 2011). As our goal was to directly test whether transportation mediates the effect of a narrative on the persuasive outcome, we made use of this relatively novel method in the present study.

### Media effects and the role of mental imagery

Although transportation is a holistic concept with cognitive, emotional, attentional, and imaginative aspects, Green and Brock (2002) particularly emphasize the importance of the vivid mental imagery that is part of transportation (hence the name Transportation *Imagery* Model). Originally, Green and Brock (2002) assumed that because it offers more opportunity for imaginative investment, “overall, reading is more likely to instigate transportation than film-viewing” (p. 330). In contrast, Green, Kass, Carrey, Herzig, Feeney, and Sabini (2008) acknowledge that text and film each have different advantages that could lead to higher transportation for one medium over the other or result in overall equal propensity to instigate transportation. The central question they highlight is whether the need to self-generate imagery in written narratives actually helps or hinders transportation. In addressing this question, it is important to consider that people differ in their ability to generate mental imagery, which could affect how well they are transported by different media (Green & Brock, 2002). Contrary to this idea, Green et al. (2008) neither found a general effect of medium on the level of transportation nor a moderating effect of imagery ability. However, they used a rather general measure of imagery ability based on items selected from Paivio’s Individual Differences Questionnaire (1971; see also Paivio & Harshman, 1983; e.g., *I find it difficult to form a mental picture of anything*), and they note that scores on this measure were generally high, suggesting that the lack of an interaction effect with medium may be due to limited variation in this variable. These limitations suggest that a more fine-grained measure that requires the active generation of mental imagery and probes the *vividness* of this imagery might better capture the underlying imagery ability that is assumed to be relevant for transportation. Therefore, we again tested Green and Brock’s (2002) and Green et al.’s (2008) prediction that imagery ability should moderate the effect of medium on transportation but with a different measure of imagery ability—namely, the *Vividness of Visual Imagery Questionnaire* (VVIQ; Marks, 1973). Although like the Individual Differences Questionnaire it is a self-report measure, it has been shown to reliably predict performance in tasks that involve visual mental imagery (e.g., Isaac & Marks, 1994; Marks, 1973; McKelvie & Demers, 1979), supporting its construct validity as a measure of the underlying ability.

### Target for persuasion: self-related control beliefs

Previous studies have shown that narratives cannot only influence recipient’s views about the external world, but also self-concept, which can be defined as “a person’s mental model of his or her abilities and attributes” (Gerrig & Zimbardo, 2002, “Self-concept”). For example, a study by Djikic et al. (2009) showed that exposure to a narrative resulted in greater change in self-reported personality traits than a non-narrative control text. Sestir and Green (2010) showed, more specifically, that temporary self-concept changes in the direction of the character traits displayed by characters in the narrative. Similarly, a study by Gabriel and Young (2011) suggested that recipients of a narrative assimilate their perception of themselves to the collective portrayed in the narrative (in this case, vampires vs. wizards), as indicated both by explicit and implicit measures. Appel (2011) found that exposure to a narrative can also prime behavior that is in line with the character traits displayed by the protagonist (i.e., a story about a stupid soccer hooligan induced worse performance in a knowledge test than a control story). Finally, Richter et al. (2014) showed that a story featuring a protagonist in a traditional gender role increased self-reported femininity in (female) recipients who were highly transported into the story and unlikely to engage in social comparison.

In sum, extant research supports the idea that narratives can at least temporarily change self-concept in the direction of the character traits displayed by central characters. The work reported here extends the research on stories and the self to the field of generalized self-related control beliefs. Specifically, we assume that they should affect recipients’ perceived general self-efficacy. This concept is based on the concept of perceived self-efficacy proposed by Bandura (1977), which refers to the expectation that one is able to carry out a particular behavior in a particular situation. Research on entertainment education (Singhal & Rogers, 1999) has aimed at positively influencing this kind of behavior-specific self-efficacy via radio or television shows that featured fictional characters carrying out a socially desirable behavior, for example, learning to read (Sabido, 1981), HIV/AIDS prevention (Vaughan, Rogers, Singhal, & Swalehe, 2000), or family planning (Rogers et al., 1999), and shown that changes in behavior-specific self-efficacy indeed contribute to performance of the targeted behavior (e.g., Papa et al., 2000). Perceived general self-efficacy, in contrast, is not situation- or behavior-specific but a generalized belief of being able to handle difficult situations (Jerusalem & Schwarzer, 1999). To our knowledge, as yet no studies have tested the impact of narratives on this kind of belief, although most narratives explicitly or implicitly convey messages regarding this domain. More specifically, many popular narratives revolve around the theme of a hero facing obstacles and adversities to come out on top in the end and be rewarded for their efforts, conveying the idea that even in adverse circumstances, one can influence one’s situation by one’s actions and choices and thereby influence one’s own fortune in this world. We assume that such narratives should, at least temporarily and to some degree, positively affect recipients’ own generalized self-related control beliefs, provided they are sufficiently transported by the narrative.

### Role of identification in narrative persuasion

Research has provided evidence for another mediator of narrative persuasion, namely the identification with central characters (De Graaf, Hoeken, Sanders, & Beentjes, 2012; Kaufman & Libby, 2012). A study by Sestir and Green (2010) even suggests that when it comes to narrative impact on self-related beliefs, identification with characters may play an even more important role than transportation into the story. According to Cohen (2001), identification means adopting the perspective of a character and feeling with the character. Oatley (1994), more specifically, proposes that this occurs by adopting the character’s goals and plans and experiencing the emotions that result from the impact of events in the story on those goals and plans. Simultaneously, identification is usually assumed to involve a loss of self-awareness. Some studies also suggest that recipients adopt the physical perspective of characters (e.g., Brunyé, Ditman, Giles, Holmes, & Taylor, 2016; Horton & Rapp, 2003), but it is unclear whether this is a necessary component of identification, and evidence suggests that a recipient’s understanding of the narrative does not become fully constrained by the perspective (physical or otherwise) of the character with whom he or she identifies (e.g., Albrecht, O’Brien, Mason, & Myers, 1995; Magliano, Taylor, & Kim, 2005; O’Brien & Albrecht, 1992). In the present study, we conceptualize identification according to Oatley’s (1994) definition and thus focus on recipients’ perspective-taking in terms of adopting the protagonist’s goals and emotionally reacting to story events depending on how they relate to those goals.

As Moyer-Gusé (2008), Sestir and Green (2010), and Tal-Or and Cohen (2016) point out, the concepts of transportation and identification are similar in that they assume a shift of the frame of reference on a cognitive, emotional, and attentional dimension. However, what differentiates the two is that identification is focused specifically on characters rather than on the narrative itself. Therefore, Sestir and Green (2010) conclude that “While the two are hardly orthogonal, and often are seen in concert, each operates independently. Thus, identification and transportation may independently and/or additively lead to activation of character traits within the self-concept of the viewer” (p. 276). In line with this notion, Tal-Or and Cohen (2016) conclude based on a review of existing studies that transportation and identification have both shared and distinctive antecedents and consequences and both clearly matter for narrative persuasion, yet “a clear answer as to how they matter is still far off” (p. 52). For this reason, in the present study we measured identification as an additional potential moderator and mediator of narrative impact.

### Study overview and predictions

The goal of the present study is to test the basic idea that narratives with strong protagonists can positively influence recipients’ self-related control beliefs against the theoretical backdrop of the Transportation Imagery Model (Green & Brock, 2002). In Experiment 1 transportation and identification are tested as potential *moderators* of the effect of stories with strong versus weak protagonists on self-related control beliefs, and (perceived) mental imagery ability is tested as a potential moderator of the effects of the medium (text vs. film) on transportation and identification. In Experiment 2 transportation and identification are manipulated experimentally and tested as potential *mediators* of the effect of a story with a strong protagonist on self-related control beliefs.

## Experiment 1

In a first experiment we tested transportation and identification as potential *moderators* of narrative impact. Participants either read or watched three stories each portraying protagonists displaying high versus low self-efficacy. We measured participants’ own perceived general self-efficacy at least one week before and again after exposure to the narratives. We also assessed their transportation and their identification with the protagonist after each narrative. In addition, we measured their perceived ability to generate vivid mental imagery and tested it as a potential moderator of media effects on transportation and identification.

In sum, we tested the following moderation hypotheses regarding the influence of medium and imagery ability on transportation and identification:

Hypothesis 1: Imagery ability moderates the effect of medium on transportation. Individuals low in imagery ability should be more transported by films than texts, whereas this difference should be reduced or absent for individuals high in imagery ability.

Hypothesis 2: Imagery ability moderates the effect of medium on identification. Individuals low in imagery ability should identify more with protagonists in films than in texts, whereas this difference should be reduced or absent for individuals high in imagery ability.

In addition, we tested the following moderation hypotheses regarding the influence of the self-efficacy manipulation, transportation, and identification on changes in participants’ reported self-efficacy:

Hypothesis 3: Transportation moderates the effect of portrayed self-efficacy on changes in reported self-efficacy. The story influence should be greater for those participants who were more deeply transported into the story worlds.

Hypothesis 4: Identification moderates the effect of portrayed self-efficacy on changes in reported self-efficacy. The story influence should be greater for those participants who identified more strongly with the protagonists of the stories.

### Methods

#### Design

The design of Experiment 1 was a 2 × 2 between-subjects design with the independent variables *portrayed self-efficacy* (high vs. low) and *medium* (written text vs. film). The dependent variables were transportation, identification, and the persuasive effect of the narrative operationalized via pre-to-post differences in participants’ self-reported general self-efficacy. Participants’ perceived ability to generate vivid mental imagery was included as a potential moderator of effects on transportation and identification. Transportation and identification, in turn, were included as potential moderators of effects on the persuasive outcome.

#### Participants

Participants were recruited from the local pool of psychology students (who participated for course credit) and from a pool of volunteers from various educational and occupational backgrounds (who participated without reimbursement). Ninety-four participants took part in the pretest that consisted of completing an online questionnaire. Of these, 88 completed the experiment proper as well. Twelve of these participants were excluded because they were either non-native speakers of German (*n* = 3) or reported not having properly followed the instructions (*n *= 8). Thus, the final sample comprised 77 participants. Of these, 55 were women, 21 were men, and 1 did not report gender. Twenty-seven of them were students (of which 20 were psychology students). Their average age was 34.4 (*SD* = 14.6) years, ranging from 15 to 69. Written parental consent was obtained for underage participants before the study. According to a sensitivity analysis computed with G*Power (Faul, Erdfelder, Lang, & Buchner, 2007), the sample size was sufficient to detect medium-sized (*f *= .32) main effects and interactions at an alpha level of .05 with a power of .80 given our design.

#### Material^1^

For the manipulation of the independent variable *portrayed self-efficacy*, we chose three narratives that portrayed protagonists displaying high self-efficacy (*Pocahontas, Merida, Beauty and the Beast*) and three narratives that portrayed protagonists displaying low self-efficacy (*Cinderella, Rapunzel, Snow White*). Three narratives each were selected instead of only one to increase the strength of the manipulation. The narratives were excerpts from Disney movies (around 10 minutes each) or the corresponding books (around 1000 words each), with comparable content in the text and video version of each narrative. The excerpts were selected in such a way that the protagonists’ self-efficacy was clearly conveyed. To match the content across media, the same scenes were selected in both the text and film versions. In addition, if the scenes contained songs in the film version, the lyrics were transcribed and integrated into the prose.

The narratives in the high self-efficacy condition all featured (female) protagonists overcoming adversities and standing up for themselves against others. In contrast, the narratives in the low self-efficacy condition featured (female) protagonists displaying passive and submissive behavior. An example that illustrates the manipulation is provided in Table A1 in the appendix.

##### Scales

The following scales were used in Experiment 1.

##### Self-efficacy

Participants’ general perceived self-efficacy was measured via the scale developed by Jerusalem and Schwarzer (1999). This scale contains 10 items that capture the general optimistic belief in oneself to cope even with difficult situations. In the original version, participants rate each item on a 4-point scale. In the present study, we used a 7-point scale instead (ranging from 1 = *not at all* to 7 = *very much*) to make the scale more sensitive to the subtle changes we expected. Participants’ baseline self-efficacy ratings were measured via an online questionnaire at least one week before exposure to the narratives; post-treatment self-efficacy was measured at the end of the second part of the experiment. Changes in self-efficacy were computed by subtracting the prescores from the postscores, with positive differences indicating an increase in self-efficacy.

##### Perceived imagery ability

Participants’ perceived ability to generate vivid mental imagery was measured in the first part of the experiment via the VVIQ (Marks, 1973). This questionnaire consists of 16 items describing situations that are supposed to evoke visual mental images in the participant (e.g., *The sun is rising above the horizon into a hazy sky*). The participant is asked to rate for each item on a scale of 1 (*No image at all, you only “know” that you are thinking of an object)* to 5 (*Perfectly clear and as vivid as normal vision*) how vivid the evoked image was in their mind. First, the participant is asked to imagine all 16 items with their eyes open and then (on a separate page) all 16 items again with their eyes closed. The results of these ratings are summed up to form a single score, but the two conditions can also be considered separately, as McKelvie (1995) has suggested that their predictive potential may diverge depending on whether the criterion task is carried out with eyes open or closed. Generally, however, “the distributions for open and closed eyes are highly correlated and similar” (Marks, 1999, p. 572). For the purpose of the present study, the questionnaire was translated from English into German.

##### Transportation

Transportation was measured with the German version of the Transportation Scale–Short Form (Appel et al., 2015) after each story. This scale consists of six items on which the participant rates his or her degree of transportation into the story (e.g., *I could picture myself in the scene of the events described in the narrative*.) on a scale from 1 (*not at all*) to 7 (*very much*). The two items capturing the imaginative component of transportation (*While reading the narrative I had a vivid image of [character name]*) were adapted to each narrative by inserting the names of the protagonist and the second most important character. In addition, some of the item wordings of the original scale refer to reading. Thus, four of the items were rephrased to match the video context. The items of both versions of the scale are displayed in both German and English in Table A2 in the appendix.

##### Identification

We assumed that the degree of identification with the protagonists of the narratives might also be a decisive factor. Therefore, we adapted items used by Sestir and Green (2010; based on items by Cohen, 2001) to assess participants’ identification on a 7-point scale ranging from 1 (*not at all*) to 7 (*very much*). The three items of the scale (translated to English) wereas follows: (1) *When good things happened to [protagonist name], I felt happy, but when negative things happened to [protagonist name], I felt sad*; (2) *When I [read the text/watched the clip], I felt or reacted as if the experiences of [protagonist name] were happening to me*; and (3) *When I [read the text/watched the clip], I wanted [protagonist name] to succeed in achieving his/her goals*.

##### Trait empathy

As participants’ trait empathy might also moderate narrative impact, we assessed it as a control variable by means of the interpersonal reactivity index (Davis, 1980, 1983), in a German adaptation by Paulus (2009). This scale assesses multiple dimensions of trait empathy, namely perspective taking, fantasy, empathic concern, and personal distress.

#### Procedure

Participants completed the first part of the experiment online from home. In this part, participants provided their informed consent, demographic data, and created an individual code to match their data from both parts. Participants were also asked about their media preference: *What would you prefer if you could choose between the same story in book versus text form?* They were also asked how many hours per week they on average spend reading books and watching movies (0–2, 2–4, 4–6, 6–8, or more than 8 hours). In addition, they were asked to indicate which “media type” they were, with the options being book type, film type, or both/neither. Subsequently, they were asked to fill out the questionnaires on empathy, on self-efficacy, and on perceived mental imagery ability. This part of the study took about 30 minutes.

Around 1 week later each participant received an invitation to the second part of the experiment, which took place in the lab. Participants were tested in groups of up to four per session. Upon their arrival, participants were randomly assigned to one of the four combinations of portrayed self-efficacy (high vs. low) and medium (text vs. film), albeit with the constraint that all participants within the same session were assigned to the same medium, to avoid drawing attention to this manipulation.

In the text condition, participants were asked to take their time and read “like you normally would”. They were then presented with the three narratives in text form (all within the same level of portrayed self-efficacy) in randomized order on the computer. Each narrative was presented in three parts to avoid participants having to scroll a lot. After each narrative, transportation and identification were measured. Then, participants were asked to indicate whether they had read the story conscientiously and normally and whether they had been familiar with the story prior to reading. This procedure was repeated for each narrative. Afterward, self-efficacy was measured again with the same scale as in the first part of the study. Finally, to match the time the session took between the text and the film condition, participants in the text condition received a number of distractor tasks and questionnaires, which were not part of the analysis.

In the film condition, participants were instructed to watch the clips attentively. They were then presented with the three narratives in video form (all within the same level of portrayed self-efficacy) in randomized order on a computer screen. After each narrative, transportation and identification were measured. Then, participants were asked to indicate whether they had watched the clip attentively and whether they had been familiar with the story prior to watching. This procedure was repeated for each narrative. Afterwards, self-efficacy was measured again with the same scale as in the first part of the study.

At the end of the session, participants were asked what they thought the aim of the study was and given the opportunity to leave comments. They were then thanked and debriefed. In total, this second part of the experiment took about an hour.

### Results

Transportation and identification were averaged across the three narratives for each participant. Pre-to-post differences in self-efficacy were computed by subtracting the prescores from the postscores (Tables 1 and 2), such that positive values indicate an increase in self-efficacy. Means, standard deviations, and intercorrelations of all covariates and dependent variables are displayed in Table 3. For all significance tests, the alpha level was set at .05. Ninety-five percent confidence intervals for Pearson correlations were computed using the SPSS Confidence Intervals for Correlations Tool (SPSS Tutorials, 2018); 90% confidence intervals for effect sizes, as recommended by Wuensch (2009) for ANOVA effects at an alpha level of .05, were computed using the effect size calculator by Uanhoro (2018). ANCOVAs were conducted with *z*-standardized covariates and interaction terms of all included variables (Aiken & West, 1991).

**Table 1.** Estimated means of reported self-efficacy at T1 and T2 as a function of transportation and portrayed self-efficacy

Transportation	Portrayed Self-Efficacy	*M_T1_*	*SE_T1_*	*M_T2_*	*SE_T2_*	*M_T2-T1_*	*SE_T2-T1_*
Low (−1 *SD*)	Low	4.68	0.15	4.81	0.14	0.13	0.11
	High	5.08	0.19	5.14	0.18	0.06	0.14
Mean	Low	4.88	0.11	4.92	0.10	0.04	0.08
	High	4.95	0.12	5.14	0.11	0.19	0.09
High (+ 1 *SD*)	Low	5.09	0.16	5.04	0.15	−0.05	0.11
	High	4.82	0.17	5.15	0.16	0.33	0.12

*Note*. Reported self-efficacy was measured on a 7-point scale (*min* = 1, *max* = 7).

**Table 2.** Estimated means of reported self-efficacy at T1 and T2 as a function of identification and portrayed self-efficacy

Identification	Portrayed Self-Efficacy	*M_T1_*	*SE_T1_*	*M_T2_*	*SE_T2_*	*M_T2-T1_*	*SE_T2-T1_*
Low (−1 *SD*)	Low	4.63	0.16	4.77	0.15	0.14	0.12
	High	4.99	0.16	5.13	0.15	0.13	0.12
Mean	Low	4.86	0.11	4.91	0.10	0.06	0.08
	High	4.94	0.12	5.15	0.11	0.21	0.09
High (+ 1 *SD*)	Low	5.08	0.15	5.06	0.14	−0.02	0.11
	High	4.88	0.18	5.17	0.17	0.30	0.13

*Note*. Reported self-efficacy was measured on a 7-point scale (*min* = 1, *max* = 7).

#### Hypothesis 1: perceived imagery ability moderates the effect of medium on transportation

**Figure 1.**
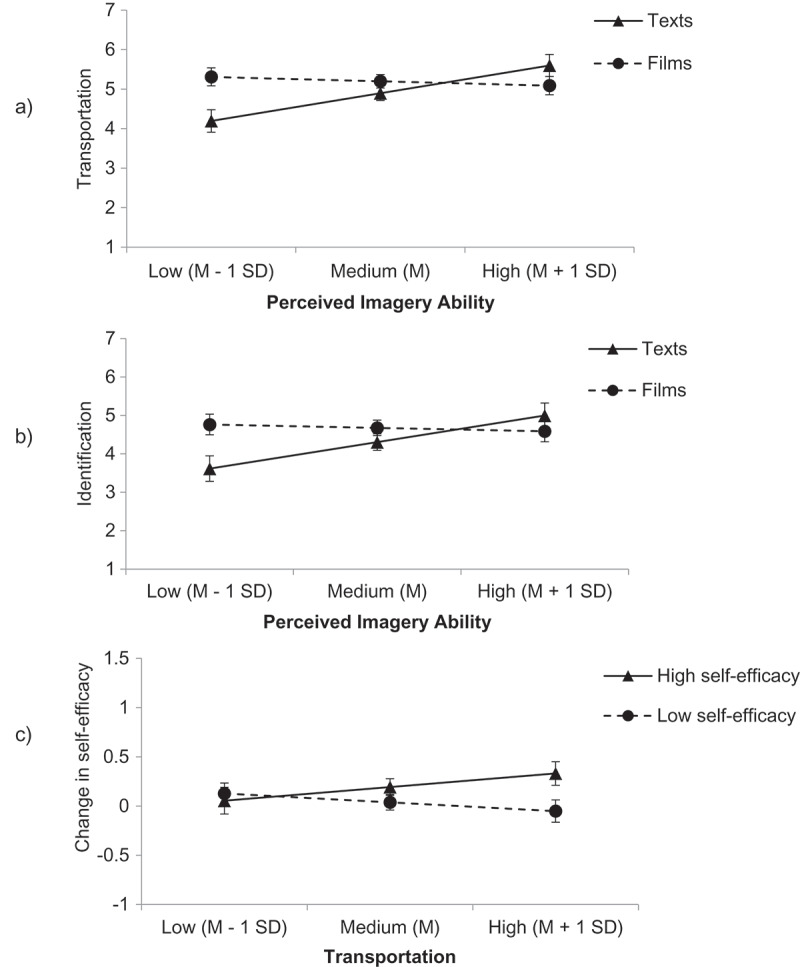
Experiment 1. (a) Transportation as a function of medium (texts vs. films) and perceived imagery ability. (b) Identification as a function of medium (texts vs. films) and perceived imagery ability. (c) Change in self-efficacy as a function of self-efficacy displayed by protagonists (high vs. low) and transportation into the stories. (Displayed are the simple slopes of the moderator in the text and the film condition. Error bars represent the standard error of the mean for the point estimates of condition means at a low, medium, or high level of the moderator variable.)

In an ANCOVA with portrayed self-efficacy and medium as independent variables and *z*-standardized perceived imagery ability as covariate, we found an interaction of medium with participants’ perceived ability to generate mental imagery on transportation, *F*(1,69) = 9.493, *p *= .003, η_p_^2^ = .121, 90% *CI* [.026, .243] (Figure 1a). We interpreted the interaction by estimating the effects of portrayed self-efficacy at a high (1 *SD* above the sample mean) and a low level (1 *SD* below the sample mean) of transportation (Aiken & West, 1991). Participants with low VVIQ scores (1 *SD* below the mean) were more transported by films (*M* = 5.310, *SE* = 0.228) than by texts (*M* = 4.194, *SE* = 0.283), *F*(1, 69) = 9.397, *p* = .003, η_p_^2^ = .120, 90% *CI* [.025, .241], whereas participants with high VVIQ scores (1 *SD* above the mean) were equally transported into texts and films (texts: *M* = 5.600, *SE* = 0.278, films: *M* = 5.090, *SE* = 0.234), *F*(1, 69) = 1.970, *p* = .165, η_p_^2^ = .028, 90% *CI* [0, .116]. In addition, there was a positive main effect of perceived imagery ability, *F*(1, 69) = 5.060, *p* = .028, η_p_^2^ = .068, 90% *CI* [.004, .178]. There were no other significant effects, all *p* ≥ .075.

#### Hypothesis 2: perceived imagery ability moderates the effect of medium on identification

In an ANCOVA with portrayed self-efficacy and medium as independent variables and *z*-standardized perceived imagery ability as covariate, we found an interaction of medium with participants’ perceived ability to generate mental imagery on identification, *F*(1,69) = 6.321, *p *= .014, η_p_^2^ = .084, 90% *CI* [.009, .198] (Figure 1b), Participants with low VVIQ scores (1 *SD* below the mean) identified more strongly with protagonists in films (*M* = 4.763, *SE* = 0.268) than in texts (*M* = 3.616, *SE* = 0.332), *F*(1, 69) = 7.232, *p* = .009, η_p_^2^ = .095, 90% *CI* [.014, .212], whereas participants with high VVIQ scores (1 *SD* above the mean) identified equally strongly with protagonists in both media (texts: *M* = 4.997, *SE* = 0.326, films: *M* = 4.589, *SE* = 0.274), *F*(1, 69) = 0.917, *p* = .342, η_p_^2^ = .013, 90% *CI* [0, .087]. No other effects were significant in this analysis, all *p* ≥ .074.

#### Hypothesis 3: transportation moderates the effect of portrayed self-efficacy on changes in reported self-efficacy

In an ANCOVA with portrayed self-efficacy and medium as independent variables and *z*-standardized transportation as covariate, we found a marginally significant interaction of portrayed self-efficacy with transportation on participants’ pre-to-post differences in self-efficacy, *F*(1,69) = 3.450, *p *= .068, η_p_^2^ = .048, 90% *CI* [0, .148] (Figure 1c). Planned contrasts showed that for participants who reported being highly transported into the narratives, self-efficacy was significantly more positively affected by the portrayal of high self-efficacy (*M* = 0.331, *SE* = 0.120) compared with the portrayal of low self-efficacy (*M* = −0.051, *SE* = 0.113), *F*(1,69) = 5.376, *p *= .023, η_p_^2^ = .072, 90% *CI* [.005, .183]. For participants who reported low transportation, there were no significant differences between the two self-efficacy conditions (high self-efficacy: *M* = 0.055, *SE* = 0.136; low self-efficacy: *M* = 0.128, *SE* = 0.106), *F*(1, 69) = 0.179, *p* = .673, η_p_^2^ = .003, 90% *CI* [0, .053]. No other effects were significant in this analysis, all *p* ≥ .17.

#### Hypothesis 4: identification moderates the effect of portrayed self-efficacy on changes in reported self-efficacy

An ANCOVA with portrayed self-efficacy and medium as independent variables and *z*-standardized identification as covariate did not yield a significant interaction of portrayed self-efficacy with identification on participants’ pre-to-post differences in self-efficacy, *F*(1,69) = 1.776, *p =* .187, η_p_^2^ = .025, 90% *CI* [0, .111], nor any other significant effects, all *p* ≥ .18.

##### Exploratory analyses

Separate ANCOVAs for transportation and identification with each of the two subscales of the VVIQ as covariate replicated the findings with the total score: For transportation, the interaction of medium with perceived imagery ability was significant both for the subscale with eyes open, *F*(1, 69) = 6.179, *p* = .015, η_p_^2^ = .082, 90% *CI* [.009, .196], and for the subscale with eyes closed, *F*(1, 69) = 4.435 *p* = .039, η_p_^2^ = .060, 90% *CI* [.002, .167]. In contrast, the interaction of medium with perceived imagery ability on identification was neither significant with eyes open, *F*(1, 69) = 2.737, *p* = .103, η_p_^2^ = .038, 90% *CI* [0, .134], nor with eyes closed, *F*(1, 69) = 3.460, *p* = .067, η_p_^2^ = .048, 90% *CI* [0, .148].

###### Media preference and perceived imagery ability

To investigate whether participants’ media preference is related to their perceived imagery ability, we ran exploratory ANOVAs with participants’ response to the question of whether they would prefer the same story in book or film format as independent variable and each of the VVIQ scores as dependent variable. Participants’ choice between book and film did not have a significant effect on their overall VVIQ score, *F*(1, 75) = 3.312, *p* = .073, η_p_^2^ = .042, 90% *CI* [0, .136], nor on their VVIQ score with eyes closed, *F*(1, 75) = 0.659, *p* = .420, η_p_^2^ = .009, 90% *CI* [0, .072], but it did on their VVIQ score with eyes open, *F*(1, 75) = 6.661, *p* = .012, η_p_^2^ = .082, 90% *CI* [.010, .190]. Participants who chose the book format (*M* = 58.447, *SE* = 1.422) had significantly higher scores than participants who chose the film format (*M* = 52.567, *SE* = 1.780). Descriptively, a similar pattern emerged for participants’ response to the question as what media type they would classify themselves (book type vs. film type vs. neither/both), with higher VVIQ scores for book types than film types and the neither/both type in between them. However, the effect of media type was not significant for any of the scores, with all *p* ≥ .054. Finally, correlating the time participants reportedly spent watching films or reading books, respectively, with each of the VVIQ scores revealed a significant correlation only between the amount of time spent reading books and the VVIQ score with eyes open, Spearman’s ρ = .376, *p* = .001, 95% *CI* [.167, .553] (confidence interval computed using the online tool provided by Lowry, 2018).

###### Effect of empathy on narrative impact

In addition, we tested whether general empathy, as measured by the interpersonal reactivity index, might also serve as a moderator of narrative impact. In contrast to this notion, an ANCOVA with portrayed self-efficacy and medium as independent variables and *z*-standardized empathy as covariate did not yield a significant interaction of portrayed self-efficacy and empathy, *F*(1, 69) = 2.236, *p* = .139, η_p_^2 ^= .031, 90% *CI* [0, .122], nor did analyses with any of the subscales as covariates (empathic concern: *F*(1, 69) = 1.207, *p* = .276, η_p_^2 ^= .017, 90% *CI* [0, .096]; fantasy: *F*(1, 69) = 0.691, *p* = .409, η_p_^2 ^= .010, 90% *CI* [0, .079]; personal distress: *F*(1, 69) = 3.288, *p* = .074, η_p_^2 ^= .045, 90% *CI* [0, .145]; perspective taking: *F*(1, 69) = 0.049, *p* = .825, η_p_^2 ^= .001, 90% *CI* [0, .035]). Instead, there was a significant but unexpected interaction of medium and empathy, *F*(1, 69) = 4.261, *p* = .043, η_p_^2^ = .058, 90% *CI* [.001, .164]. This interaction was driven by the fact that highly empathic participants (1 *SD* above the mean) were significantly more positively affected in their self-efficacy by films (*M* = 0.407, *SE* = 0.111) than texts (*M* = 0.051, *SE* = 0.122) regardless of portrayed self-efficacy condition, *F*(1, 69) = 4.676, *p* = .034, η_p_^2^ = .063, 90% *CI* [.003, .171].

### Discussion

To summarize, transportation was found to be a marginally significant moderator of narrative impact, whereas identification was not. Moreover, the perceived ability to generate visual mental imagery could be shown to be an important moderator of media effects on both transportation and identification: Whereas participants high in imagery ability reported equal transportation and identification for both media, participants low in imagery ability reported less transportation and identification for texts than films, indicating that they benefited from the visually provided imagery in films. Exploratory analyses suggested that particularly the ability to generate mental imagery with eyes open is related to a preference for and exposure to written narratives.

The finding that transportation moderates narrative impact confirms the predictions of the Transportation Imagery Model by Green and Brock (2002). However, a more convincing case for a causal role of transportation in narrative persuasion could be made if an experimental manipulation of transportation could be shown to directly affect the persuasive outcome. This was our goal in Experiment 2.

## Experiment 2

To further test the assumption that transportation is crucial for the effect of stories on self-related control beliefs, we varied transportation into the story world experimentally by means of a review treatment before the presentation of the narrative (e.g., Gebbers et al., 2017; Shedlosky-Shoemaker et al., 2011; see also Tukachinsky, 2014) and tested (experienced) transportation and identification as potential *mediators* of the effect of a story on self-related control beliefs. Given this focus on mediation in Experiment 2, we dropped the experimental variation of medium and only used a film in this study, as this medium had turned out to transport recipients more consistently in Experiment 1. Again, we measured participants’ self-efficacy at least 1 week before and again after exposure to the narrative. In this study, we additionally measured the related construct of *internal control beliefs* for a broader spectrum of self-related control beliefs that might be affected by narratives with strong protagonists. According to Rotter (1966), individuals develop generalized expectancies regarding the locus of control of reinforcement. An individual with *external control beliefs* perceives reinforcements to be outside of their own control and to depend instead on chance or powerful others. In contrast, an individual with *internal control beliefs* perceives reinforcements to be generally contingent on their behavior and therefore controllable. Although the concept of self-efficacy originally refers to beliefs regarding the ability to perform certain actions, whereas locus of control beliefs concern the *instrumentality* of one’s actions, in their generalized forms they capture different aspects of the belief that one controls one’s own life, and thus both could be useful to capture the persuasive impact of narratives with strong protagonists.

For the manipulation check, we tested

Hypothesis 5: The review treatment has an effect on experienced transportation and identification. Participants who read a positive review before watching the movie should report higher transportation and identification than participants who read a negative review.

A successful manipulation of transportation and identification should in turn influence the persuasive outcome, which results in

Hypothesis 6: The review treatment has an effect on the persuasive outcomes. Participants who read a positive review before watching the movie should exhibit a more positive shift in their self-efficacy and internal control beliefs than participants who read a negative review.

Finally, we ran mediation analyses to test whether the persuasive impact of the narrative was indeed mediated by experienced transportation, identification, or both, testing the following hypotheses:

Hypothesis 7: Transportation mediates narrative impact. Participants’ positive shifts in their self-efficacy and internal control beliefs should be partially or fully mediated by their experienced transportation.

Hypothesis 8: Identification mediates narrative impact. Participants’ positive shifts in their self-efficacy and internal control beliefs should be partially or fully mediated by their experienced identification with the protagonist of the movie.

### Methods

#### Design

The design was a one factorial between-subjects design with the two-level factor *review treatment* (positive vs. negative review) meant to induce different levels of transportation (and, perhaps, identification). The dependent variables were the pre-to-post differences in participants’ self-efficacy and internal control beliefs. As potential mediators, experienced transportation and identification were included in the design.

#### Participants

Participants were recruited from the local pool of psychology students (who participated for course credit) and from a pool of volunteers from various educational and occupational backgrounds (who participated without reimbursement). Sixty-three participants took part in the online pretest. Of these, 57 participated in the experiment proper as well. One participant was excluded from the analysis because she already knew the film used in the study. Thus, the final sample comprised 56 participants, with 28 in each of the two conditions (positive vs. negative review). Of these, 38 were female and 18 were male. Fifty of them were students (of which 43 were psychology students). Their average age was 24.59 (*SD* = 5.24) years, ranging from 19 to 40 years. Most of the participants (52) were native speakers of German. The four non-native speakers were not excluded from the sample as the audiovisual narrative used in this study was assumed to require less proficiency in German than a written narrative. According to a sensitivity analysis computed with G*Power (Faul et al., 2007), the sample size was sufficient to detect medium-sized (*f *= .38) main effects and interactions at an alpha level of .05 with a power of .80 given our design.

#### Material^1^

For this study we chose a film that, like the narratives portraying highly self-efficacious protagonists in Experiment 1, could be assumed to positively influence the recipients’ beliefs about self-efficacy and locus of control. “Butterfly Circus” is an American short film produced by Joshua and Rebekah Weigel in 2009 with a total length of 23 minutes. The main character, Will (played by motivational speaker Nick Vujicic), was born without arms and legs but overcomes the challenges of his disability to eventually become a successful circus star. The (implicit) moral of the story is that anyone can achieve success no matter how adverse the circumstances. Therefore, we assumed the story to have a positive impact on recipients’ self-related control beliefs. As no dubbed version was available, an English version with German subtitles was used in this study.

##### Experimental manipulation of transportation

To manipulate transportation (and, potentially, identification as well), we used two different reviews, ostensibly from a website about new movie releases (cinema.de). Exactly the same reviews had successfully been used to manipulate transportation in previous studies using the same short film (e.g., Bacherle, 2015). One group of participants received a negative review before watching the film and the other group received a positive review. The positive review praised various aspects of the film that suggested a high potential for transportation (e.g., convincing acting performances, suspense, emotionally touching story and characters). The negative review, in contrast, contained negative evaluations of the same aspects, suggesting a low potential for transportation.

##### Scales

The following scales were used in Experiment 2.

###### Transportation

The same adapted version of the Transportation Scale–Short Form (Appel et al., 2015) was used as in Experiment 1.

###### Identification

The same identification scale based on Sestir and Green (2010) was used as in Experiment 1.

###### Self-efficacy

The self-efficacy scale developed by Jerusalem and Schwarzer (1999) was used as in Experiment 1. However, in the present study, the scale was used in its original form, that is, with a 4-point (instead of a 7-point) response scale offering the response option “strongly disagree”, “disagree”, “agree”, and “strongly agree”.

###### Internal control beliefs

In this experiment we measured an additional variable related to self-efficacy that we assumed might also be able to capture the persuasive impact of the selected narrative, namely the IPC scales by Levenson (1974), in a German adaptation by Krampen (1979). This questionnaire consists of 24 items, of which eight items each form a subscale. For the study at hand we focused on the I-Scale, which consists of items that refer to an internal attribution of control over life events. The remaining two subscales measure external (P-Scale) and external-fatalistic attributions of control (C-Scale) and were not relevant for the hypotheses. Accordingly, they were not included in the analyses. The items were rated on a 6-point scale, with the response options ranging from “strongly disagree” to “strongly agree”.

#### Procedure

As in Experiment 1, participants completed the first part of the experiment as an online survey at home. First, they were asked to provide informed consent, demographic data, and to generate an individual code to match the data of both parts of the experiment. The survey continued with the scales for self-efficacy and locus of control to measure participants’ baseline scores. At the end, participants were thanked and asked to sign up for the second part of the study 1 to 2 weeks later. This part of the experiment took approximately 10 minutes.

The second part of the experiment took place in the lab. Participants were tested in groups of up to four per session. Upon their arrival, participants generated their individual code again and were assigned to one of the two review treatment conditions (positive vs. negative). Their baseline scores of self-efficacy and internal locus of control were used to randomly assign participants to the conditions in matched pairs and thereby guarantee nearly identical baselines for both groups to control for potential confounds (matched groups design; e.g., Bortz & Döring, 2006; Shaughnessy, Zechmeister, & Zechmeister, 2003). Depending on their assignment, they read either the positive or the negative review. Then, they watched the short film “Butterfly Circus”. Subsequently, experienced transportation and identification were measured; afterward, post-treatment self-efficacy and internal control beliefs were assessed. To ascertain whether participants had watched the film attentively they were asked to answer seven multiple-choice comprehension questions (e.g., *What kind of disability does Will have?*) and to summarize the moral of the story. In addition, they were asked if they already knew the film, how realistic they found it, and how they evaluated it. At the end, they were asked if they believed that the film had influenced the way they answered the questions afterward and were given the opportunity to leave comments. Finally, they were thanked and debriefed. This second part of the study took approximately 35 to 45 minutes.

### Results

Pre-to-post differences in self-efficacy and internal control beliefs were computed by subtracting the prescores from the postscores (Table 4), such that positive values indicate an increase. Means, standard deviations, and intercorrelations of all mediators and dependent variables are displayed in Table 5. For all significance tests, the alpha level was set at .05. Ninety-five percent confidence intervals for Pearson correlations were computed using the SPSS Confidence Intervals for Correlations Tool (SPSS Tutorials, 2018); 90% confidence intervals for effect sizes, as recommended by Wuensch (2009) for ANOVA effects at an alpha level of .05, were computed using the effect size calculator by Uanhoro (2018). ANCOVAs were conducted with *z*-standardized covariates and interaction terms of all included variables (Aiken & West, 1991).

**Table 3.** Means, standard deviations, and intercorrelations of covariates and dependent variables in Experiment 1

Variable	*M*	*SD*	1	2	3	4	5	6
1. Change in self-efficacy	0.12	0.50	–					
2. Transportation	5.07	1.17						
*r*			.008	–				
*p*			.948					
95% *CI*			−.22, .23					
3. Identification	4.54	1.34						
*r*			−.024	.881	–			
*p*			.839	< .001				
95% *CI*			−.25, .20	.82, .92				
4. VVIQ Total	112.00	20.99						
*r*			−.102	.152	.146	–		
*p*			.380	.188	.205			
95% *CI*			−.32, .12	−.07, .36	−.08, .36			
5. VVIQ Eyes Open	56.16	10.11						
*r*			−.214	.119	.065	.726	–	
*p*			.062	.305	.574	< .001		
95%*CI*			−.42, .01	−.11, .33	−.16, .28	.60, .82		
6. VVIQ Eyes Closed	55.84	15.32	***					
*r*			−.002	.129	.157	.891	.335	–
*p*			.986	.262	.173	< .001	.003	
95% *CI*			−.22, .22	−.10, .34	−.07, .37	.83, .93	.12, .52	
7. Empathy	3.26	0.38						
*r*			.127	.389	.380	.303	.232	.226
*p*			.273	< .001	.001	.007	.042	.049
95% *CI*			−.10, .34	.18, .56	.17, .55	.08, .49	.01, .43	.00, .43

*Note. N* = 77.

**Table 4.** Estimated means of reported self-efficacy and internal control beliefs at T1 and T2 as a function of review treatment

Dependent Variable	Review Treatment	*M_T1_*	*SE_T1_*	*M_T2_*	*SE_T2_*	*M_T2-T1_*	*SE_T2-T1_*
Reported self-efficacy	Negative	2.99	0.06	3.07	0.07	0.08	0.05
	Positive	2.99	0.06	3.08	0.07	0.09	0.05
Internal control beliefs	Negative	4.38	0.09	4.45	0.09	0.06	0.06
	Positive	4.34	0.09	4.62	0.09	0.28	0.06

*Note*. Reported self-efficacy was measured on a 4-point scale (*min* = 1, *max* = 4), and internal control beliefs were measured on a 6-point scale (*min* = 1, *max* = 6).

**Table 5.** Means, standard deviations and intercorrelations of mediators and dependent variables in experiment 2

Variable	*M*	*SD*	1	2	3
1. Change in self-efficacy	0.08	0.28	–		
2. Change in internal control beliefs	0.17	0.31			
*r*			.184	–	
*p*			.174		
95% *CI*			−.08, .42		
3. Transportation	5.71	0.98			
*r*			.398	.392	–
*p*			.002	.003	
95% *CI*			.15, .59	.14, 59	
4. Identification	5.07	1.27			
*r*			.410	.362	.759
*p*			.002	.006	< .001
95% *CI*			.16, .60	.11, .57	.62, .85

*Note. N* = 56.

#### Hypothesis 5: manipulation check

The review treatment was successful at manipulating transportation: Participants who had received a positive review (*M* = 6.048, *SE* = 0.176) experienced significantly more transportation into the narrative than participants who had received a negative review (*M* = 5.363, *SE* = 0.176), *F*(1, 54) = 7.574, *p* = .008, η_p_^2^ = .123, 90% *CI* [.019, .260].

The review treatment also had a significant effect on identification: Participants who had received a positive review (*M* = 5.536, *SE* = 0.226) reported significantly more identification with the protagonist than participants who had received a negative review (*M* = 4.607, *SE* = 0.226), *F*(1, 54) = 8.444, *p* = .005, η_p_^2^ = .135, 90% *CI* [.024, .274].

#### Hypothesis 6: effect of manipulation on persuasive outcome

The review treatment, surprisingly, did not have a significant effect on pre-to-post differences in self-efficacy, *F*(1, 54) = 0.020, *p* = .888, η_p_^2^ = .000, 90% *CI* [0, .028]. It did, however, have a significant effect on pre-to-post differences in internal control beliefs: Participants who had received a positive review (*M* = 0.281, *SE* = 0.055) exhibited a significantly greater increase in their internal control beliefs than participants who had received a negative review (*M* = 0.063, *SE* = 0.055), *F*(1, 54) = 7.868, *p* = .007, η_p_^2^ = .127, 90% *CI* [.021, .265].

#### Hypothesis 7: experienced transportation as mediator of narrative impact

**Figure 2.**
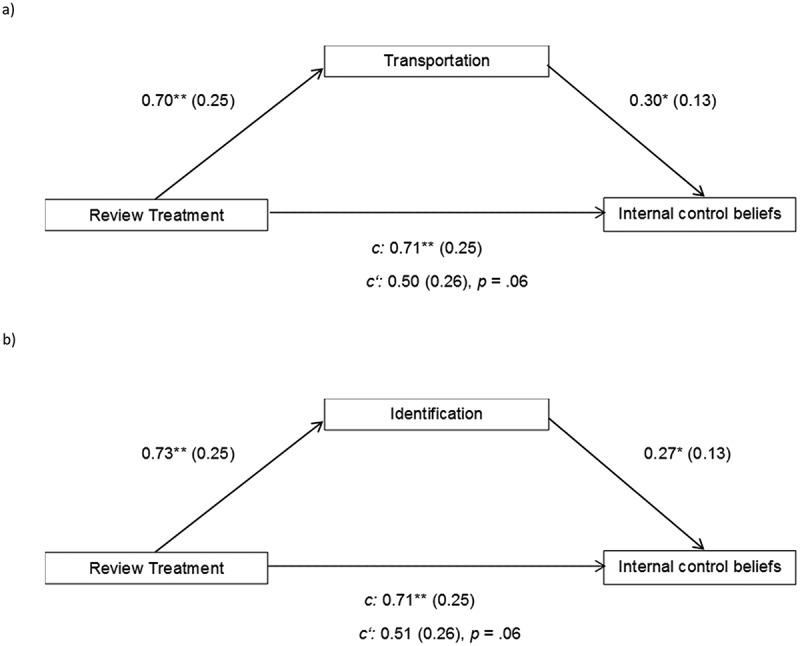
Mediation models for the indirect effect of review treatment (negative = 0, positive = 1) on pre-to-post differences in internal control beliefs via transportation (*top*) and identification (*bottom*) (all *z*-standardized).

To test whether the effect of the manipulation on the persuasive outcome was indeed mediated by the degree to which recipients were transported into the narrative, we ran mediation analyses using the PROCESS macro by Hayes (2013, 2017)). An analysis with review treatment as predictor (negative = 0, positive = 1), *z*-standardized differences in internal control beliefs as dependent variable, and *z*-standardized transportation as mediator revealed a significant indirect effect of the manipulation via transportation, *b *= .21, 95% *CI* [.024, .577] (Figure 2a).

#### Hypothesis 8: experienced identification as mediator of narrative impact

We also tested the hypothesis of a mediating role of identification. Here as well, we found a significant indirect effect of review treatment on changes in internal control beliefs via identification, *b *= .19, 95% *CI* [.005, .532] (Figure 2b).

### Discussion

The results of Experiment 2 strongly support the hypothesis that both transportation and identification play a causal role in narrative persuasion. The review treatment successfully manipulated both experienced transportation into the narrative and experienced identification with the protagonist. These variables, in turn, affected narrative impact in terms of changes in internal control beliefs (but not in self-efficacy), as could be shown by mediation analyses.

It was unexpected, however, that the results for the two dependent variables self-efficacy and internal control beliefs would diverge in this way. We had expected that both the change in self-efficacy and the change in internal control beliefs would be affected by the experimental manipulation. In contrast to this prediction, only internal control beliefs were affected. Moreover, the pre-to-post differences in both variables were not correlated (Table 5). This is surprising because when considering the individual items, the two scales appear to measure quite similar concepts. In line with this notion, the variables were significantly correlated at both times of measurement (pretest: *r* = .49, *p* < .001, 95% *CI* [.26, .67]; post-test: *r* = .60, *p* < .001, 95% *CI* [.40, .74]), and the differences in each of them were significantly related to both transportation and identification (Table 5). One possible explanation for this unexpected divergence of the two dependent variables is that the 4-point self-efficacy scale was too coarse to capture the subtle changes. Given that self-efficacy is a rather stable personality trait that is unlikely to change drastically by exposure to a single and relatively short narrative, it probably would have been better to use a more fine-grained assessment of self-efficacy, such as the 7-point response scale in Experiment 1. It is possible that only the I-scale (which had a 6-point response scale) that was used to measure internal control beliefs was able to capture the subtle changes induced by the narrative in this experiment. Another possibility is that the I-scale was more suitable to capture the aspect of self-related control beliefs that was implicated by the narrative. This highlights the importance of choosing the right dependent variables to measure the narrative impact of a particular story, even among constructs that appear to be highly similar.

## General discussion

The results of the two experiments support the applicability of the Transportation Imagery Model (Green & Brock, 2002) to persuasive influences of narratives on self-related beliefs. Specifically, they suggest that a story portraying a strong protagonist can, at least temporarily, positively affect the recipient’s own generalized self-related control beliefs. Experiment 1 showed that the persuasive impact of stories portraying low versus high self-efficacy is moderated by transportation (as suggested by a marginally significant interaction), but not by identification. Experiment 2 manipulated both transportation and identification experimentally and established them as mediators of the persuasive impact of such a story. These results support the assumption that both transportation and identification can function as mechanisms of narrative persuasion.

### Self-related control beliefs as the target for narrative persuasion

A general challenge in experimentally studying narrative persuasion is finding narratives that are short enough to be used in the lab but at the same time sufficiently persuasive to induce at least subtle changes in recipients’ attitudes, beliefs, or self-concept. The present study provides initial evidence that generalized self-related control beliefs are a promising domain for studying narrative impact, not only because they are such a common theme in popular narratives, but also because well-validated scales exist to measure them. That said, the results of our experiments highlight the importance of using fine-grained scales to capture the subtle changes that narratives can induce in these generally rather stable traits. Accordingly, the effect sizes of the obtained effects were quite small, but we would not have expected more drastic changes given the briefness of exposure.

The fact that even such a relatively short exposure to certain kinds of narratives can have (at least in the short term) differential effects on participants’ own self-related control beliefs again underlines the persuasive power inherent to narratives. Based on the results, it seems likely that more long-term and repeated exposures to narratives with protagonists displaying either high or low self-efficacy might permanently shape (or shift) recipients’ own self-related control beliefs, especially in young recipients whose personality is still in the process of developing. These hypotheses could be tested with longitudinal designs and with samples of different ages. If they are confirmed, then carefully selecting the narratives to which one exposes oneself (or, e.g., one’s children) could be an effective means of positively influencing one’s (or one’s children’s) self-related control beliefs. At the same time, this would mean that frequent exposure to narratives featuring protagonists displaying low self-efficacy—as seems to be the case for many female characters in traditional fairy-tales—could potentially have harmful effects, not only on the individual but also on society as a whole, when such narratives constitute part of society’s common ground. This underlines the importance of investigating the precise mechanisms of narrative persuasion so that, ultimately, positive effects of narratives on both the individual and on society can be maximized while negative effects can be minimized or avoided altogether.

On a more general level, the results suggest that people incorporate beliefs derived from narratives and embodied by their protagonists into their own lives, which coheres well with other findings on the persuasive effects of narratives (e.g., on self-reported femininity [Richter et al., 2014] or on affective values of children [Tsai, Louie, Chen, & Uchida, 2007]). Naturally, to maximize our chances of finding an effect from a relatively short exposure, we used narratives that quite clearly illustrated high or low self-efficacy, respectively. At the same time, it must be noted that the messages of the narratives regarding control beliefs were nonetheless implicit, communicated only via the portrayal of characters behaving in one way or another. Thus, we would assume that our findings generalize to other literature, even literature with more subtle messages. In addition, the malleability of one’s own fate—or, to put it in the words of the opening quote: the idea that “dragons can be beaten”—is a pervasive topic in literature and at the same time a fundamental belief that affects many areas of life, which is why we think the results are of high ecological relevance. Of course, experiments always entail reductions and simplifications compared to real life, but the fact the we used real narratives of high cultural relevance and popularity makes us confident in the ecological validity of our study.

### Experimental manipulation of transportation and identification

Another encouraging result of the present study is that the experimental manipulation of transportation by means of a review treatment was again successful, as in previous studies (e.g., Gebbers et al., 2017; Shedlosky-Shoemaker et al., 2011). This corroborates the notion that this elegant yet simple and universally applicable technique could be a reliable means of manipulating transportation into narratives in different media, which opens up exciting possibilities for future research on narrative persuasion. That said, it has to be noted that it is as of yet unclear which precise characteristics of the reviews make them effective—or in other words, how precisely such reviews need to be crafted to successfully manipulate experienced transportation. This is a question that will need to be addressed by future research. Another new insight from the present study is that this review treatment can also be used successfully to experimentally manipulate identification with story characters, which again opens up possibilities for further investigating the nature and consequences of this process.

### Transportation and identification

In line with previous findings (e.g., Sestir & Green, 2010) and theoretical considerations (e.g., Moyer-Gusé, 2008; Tal-Or & Cohen, 2016), our results suggest that transportation and identification, although highly related, are different mechanisms that both play an important role in narrative persuasion. Most notably, they both turned out to be sensitive to the experimental manipulation in Experiment 2 and to be mediators of narrative impact, yet only transportation was also found to be a significant moderator of narrative impact in Experiment 1. This is in line with previous findings showing that transportation and identification have both shared and distinctive antecendents and consequences (for an overview, see Tal-Or & Cohen, 2016). It also supports Sestir and Green’s (2010) proposal that transportation and identification operate independently and/or additively in narrative persuasion—probably depending both on the particular narrative and the persuasive outcome that is being investigated. It is likely that, as Tal-Or and Cohen (2016) suggest, the respective contributions of both processes to narrative persuasion depend on whether a persuasive message is conveyed more by the narrative as a whole (in terms of the “moral of the story”) or whether it is expressed by a character. In the former case, transportation should be relatively more important than identification, whereas it should be the other way around in the latter case. In the narratives we used in the present study, the most persuasive element was probably the overall structure of the narratives (with characters being rewarded in the end for being courageous and overcoming challenges and adversities) rather than the behavior or views of the characters themselves. This could explain why transportation moderated narrative impact in Experiment 1 (as suggested by a marginally significant interaction) but identification did not. In summary, the results of the present study contribute to shedding some light on the interplay of both processes, but as Tal-Or and Cohen (2016) point out, further research on this topic seems necessary to obtain a more thorough understanding.

### Role of mental imagery

As postulated by Green and Brock (2002), imagery indeed seems to play an important role in transportation. Using a more fine-grained measure of perceived imagery ability than in previous research (Green et al., 2008), the present study demonstrated that transportation into a narrative and identification with its protagonist vary across different media depending on recipients’ perceived ability to generate vivid mental imagery. More specifically, recipients with low perceived imagery ability seem to benefit from the “ready-made” imagery provided by audiovisual narratives, allowing them to be more highly transported and identify more strongly with characters than in purely written narratives. Recipients with high perceived imagery ability, in contrast, display equal transportation and identification for both texts and films, probably because the imagery they are able to generate from written text is comparable to the imagery provided visually by films. This pattern of results suggests that it is not the self-generation of imagery (or imaginative investment, as it is termed by Green & Brock, 2002) that is central to transportation, but rather the vividness of the images, regardless of whether they are self-generated or provided by the medium. Interestingly, exploratory analyses with separate scores for the perceived ability to generate mental imagery with eyes open or closed suggest that the eyes open condition is more closely related to a media preference for books, as well as to the number of hours spent reading books—which makes sense considering that when reading a narrative, imagery usually needs to be generated *at the same time* as the text is being read, that is, while the eyes are open (cp. McKelvie, 1995). This is likely due to a bidirectional relationship between preference for and time spent reading books, on the one hand, and the ability to generate imagery with one’s eyes open, on the other hand. If this assumption holds, attempting to train the skill of generating vivid mental imagery, in particular with one’s eyes open, could be a useful intervention to increase young people’s interest in books, which could in turn positively influence their overall reading skill and enjoyment. However, it must be pointed out that further studies with higher power are necessary to confirm whether indicators of perceived ability to generate mental imagery with eyes open or closed have reliably differential associations with media preference and exposure.

### Limitations

Although establishing transportation as both moderator and mediator and identification as mediator of narrative impact supports the notion of their causal role in narrative persuasion, it must be noted that the data of the present experiments do not provide proof for causality. While our successful experimental manipulation of the intervening variable and its corresponding effect on the outcome variable in Experiment 2 makes a stronger case for this assumption than the moderating effect of transportation found in Experiment 1, there is still ambiguity regarding the direction of the relationship between the mediators (transportation, identification) and the outcome variable, as both were measured after exposure to the narrative (although the mediators were measured before the outcome variable). It would be preferable in future studies to empirically test the assumed temporal sequence by measuring transportation concurrently during exposure to the narrative. Methods for doing so are being explored, but it must be noted that none of the measures used so far are able to comprehensively capture the phenomenon of transportation, only aspects of it (e.g., the attentional or emotional components; Bezdek & Gerrig, 2017; Hartung et al., 2016; Sukalla et al., 2016), and their relationships to self-reported transportation are sometimes inconsistent (e.g., Hartung et al., 2016). Nonetheless, it seems desirable for future research to further explore the usefulness of such measures and eventually overcome the limitations imposed by retrospective self-report measures of media experiences. Another (albeit related) limitation results from the strong correlation of the transportation and identification measures, which made it impossible to estimate a multiple mediation model in Experiment 2 due to concerns of multicollinearity. In principle, a multiple mediation model could shed light on the relative contribution of transportation and identification to the impact of narratives on self-related beliefs, going beyond the separate mediation models estimated in Experiment 2. However, the strong correlation between transportation and identification, which is in the magnitude of the reliabilities of the two scales, suggests that the two constructs are strongly overlapping, at least when measured with the scales and for a narrative of the kind that we used.

A limitation that must also be noted are the relatively small sample sizes of the two studies, which leave open the question of the reliability of some of the results. Most notably, this concerns the only marginally significant interaction of portrayed self-efficacy and transportation on pre-to-post changes in self-efficacy but also, for example, the differential associations of the two imagery subscales with media preference and exposure. Certainly, this study can therefore only be seen as a first step toward studying empowerment by means of narratives, and studies with larger samples are desirable in future research. In addition, there might be gender effects on transportation, identification, and persuasion that could only be uncovered by samples with equal numbers of men and women in each condition.

Finally, in Experiment 1 there is a potential confound with familiarity, as the narratives in the low self-efficacy condition were all traditional fairy-tales that are known by most Germans, whereas the narratives in the high self-efficacy condition were more modern tales and therefore overall somewhat less well known. This difference results quite naturally from the relatively recent cultural acceptance and popularity of narratives with strong female heroines, in contrast to the traditional submissive female stereotype that is prevalent in older narratives. For future research, however, it would be desirable to use narratives that are similarly familiar or unfamiliar and stem from a similar time period or to use the same narrative in different conditions (as in Experiment 2).

### Conclusion

The present study extends research on narrative persuasion to the domain of self-related control beliefs (perceived general self-efficacy and internal control beliefs), showing that they can be influenced by stories with strong protagonists managing to overcome adversities. It confirms the predictions derived from the Transportation Imagery Model (Green & Brock, 2002) that transportation plays an important role as moderator and mediator of this process. In line with Gerrig’s (1993) suggestion, recipients who are highly transported into a story do seem to return from the journey somewhat changed—in this case, with stories conveying that “dragons can be beaten”, they returned stronger. Finally, the present study sheds light on the role of mental imagery in transportation and identification in different media, and on the role of identification as an additional mediator of narrative persuasion.

## Notes

1. Most of the material described here is available in an online appendix at https://osf.io/43e9d/. In addition, all material is available from the authors on request for research purposes.

## Appendix A

**Table A1.** Illustration of the self-efficacy manipulation in Experiment 1

Condition	Example passage (German)	Example passage (translated from German)
Low portrayed self-efficacy	[…] „Rapunzel, ich habe eine Bitte“, sagte Mutter Gothel mit fester Stimme. „Ja, Mutter?“, Rapunzel blickte sie fragend an. „Bitte nie wieder darum, diesen Turm verlassen zu dürfen. Nie wieder!“, sagte Mutter Gothel mit herrischer Stimme. „Ja, Mutter.“, antwortete Rapunzel traurig.	„[…] Rapunzel, I have a request“, said Mother Gothel firmly. „Yes, Mother?“, Rapunzel looked at her inquiringly. „Never ask again to be able to leave this tower. Never!“, Mother Gothel commanded. „Yes, Mother“, Rapunzel responded sadly.
High portrayed self-efficacy	„[…] „Ich bin das erstgeborene Kind des DunBroch-Clans“, erklärte sie laut. „Und ich werde um meine eigene Hand wettstreiten!“ „Das verbiete ich!“, rief die Königin Elinor. Merida aber beachtete sie nicht und zielte.	„[…] „I am the first-born child of the DunBroch-Clan“, she said loudly. „And I will compete for my own hand in marriage!“ „I’ll forbid it!“, cried Queen Elinor. But Merida ignored her and took aim.

**Table A2.** Transportation items adapted from the Transportation Scale–Short Form (TS-SF; Appel et al., 2015).

TS-SF Item No.	Facet	TS-SF English	TS-SF German	TS-SF German (videos)	TS-SF (videos, translated into English)
1.	Cognitive	I could picture myself in the scene of the events described in the narrative.	Ich konnte mich selbst in der Szenerie sehen, die in der Geschichte beschrieben wird.	^b^Ich habe mich so gefühlt, als ob ich selbst in der Szenerie, die in der Geschichte gezeigt wird, dabei gewesen wäre.	^b^I felt as if I was present in the scene of the events portrayed in the narrative.
2.	Cognitive	I was mentally involved in the narrative while reading it.	Während des Lesens fühlte ich mich gedanklich in die Geschichte hineingezogen.	^b^Während ich den Filmausschnitt angesehen habe, fühlte ich mich gedanklich in die Geschichte hineingezogen.	^b^I was mentally involved in the narrative while watching it.
3.	General	I wanted to learn how the narrative ended.	Ich wollte wissen, wie die Geschichte ausgeht.	Ich wollte wissen, wie die Geschichte ausgeht.	I wanted to learn how the narrative ended.
4.	Emotional	The narrative affected me emotionally.	Die Geschichte hat mich emotional berührt.	Die Geschichte hat mich emotional berührt.	The narrative affected me emotionally.
5.	Imaginative	^ab^While reading the narrative I had a vivid image of [character name].	^ab^Während ich die Geschichte las, konnte ich mir [Charaktername] lebhaft vorstellen.	^b^Wenn ich an den Filmausschnitt zurückdenke, habe ich das Bild von [Charaktername] lebhaft vor Augen.	^b^When I think back on watching the video, I have a vivid image of [character name].
6.	Imaginative	^ab^While reading the narrative I had a vivid image of [character name].	^ab^Während ich die Geschichte las, konnte ich mir [Charaktername] lebhaft vorstellen.	^b^Wenn ich an den Filmausschnitt zurückdenke, habe ich das Bild von [Charaktername] lebhaft vor Augen.	^b^When I think back on watching the video, I have a vivid image of [character name].

*Note*. Items were presented with 7-point response scales from 1 (*not at all*) to 7 (*very much*).

^a^These items were adapted to the respective narrative by inserting the names of the main characters of the narrative.

^b^These items were adapted for the present study to be applicable to narratives presented in video form.

## References

[CIT0001] Aiken, L. S., & West, S. G. (1991). *Multiple regression: Testing and interpreting interactions*. Thousand Oaks, CA: Sage.

[CIT0002] Albrecht, J. E., O’Brien, E. J., Mason, R. A., & Myers, J. L. (1995). The role of perspective in the accessibility of goals during reading. *Journal of Experimental Psychology: Learning, Memory, & Cognition*, 21, 364–372. 10.1037//0278-7393.21.2.364 7738505

[CIT0003] Appel, M. (2011). A story about a stupid person can make you act stupid (or smart): Behavioral assimilation (and contrast) as narrative impact. *Media Psychology*, 14, 144–167. doi:10.1080/15213269.2011.573461

[CIT0004] Appel, M., Gnambs, T., Richter, T., & Green, M. (2015). The transportation scale – short form (TS-SF). *Media Psychology*, 18, 243–266. doi:10.1080/15213269.2014.987400

[CIT0005] Bacherle, P. (2015). Eintauchen in narrative Welten - Theoretische und empirische Zugänge zum Rezeptionserleben [Transportation into narrative worlds–Theoretical and empirical approaches to reception experiences] (Doctoral thesis). University of Koblenz-Landau, Landau, Germany. Retrieved from https://kola.opus.hbz-nrw.de/frontdoor/index/index/docId/935

[CIT0006] Bandura, A. (1977). Self-efficacy: Toward a unifying theory of behavioral change. *Psychological Review*, 84, 191–215. 847061 10.1037//0033-295x.84.2.191

[CIT0007] Bezdek, M. A., & Gerrig, R. J. (2017). When narrative transportation narrows attention: Changes in attentional focus during suspenseful film viewing. *Media Psychology*, 20, 60–89. doi:10.1080/15213269.2015.1121830

[CIT0008] Bortz, J., & Döring, N. (2006). *Forschungsmethoden und Evaluation für Human- und Sozialwissenschaftler* (4th ed.) [Electronic version]. doi:10.1007/978-3-540-33306-7

[CIT0009] Brunyé, T. T., Ditman, T., Giles, G. E., Holmes, A., & Taylor, H. A. (2016). Mentally simulating narrative perspective is not universal or necessary for language comprehension. *Journal of Experimental Psychology: Learning, Memory, and Cognition*, 42, 1592–1605. doi:10.1037/xlm0000250 26889684

[CIT0010] Cohen, J. (2001). Defining identification: A theoretical look at the identification of audiences with media characters. *Mass Communication & Society*, 4, 245–264.

[CIT0011] Davis, M. H. (1980). A multidimensional approach to individual differences in empathy. *Catalog of Selected Documents in Psychology*, 10, 85.

[CIT0012] Davis, M. H. (1983). Measuring individual differences in empathy: Evidence for a multidimensional approach. *Journal of Personality and Social Psychology*, 44, 113–126. doi:10.1037/0022-3514.44.1.113

[CIT0013] De Graaf, A., Hoeken, H., Sanders, J., & Beentjes, H. (2009). The role of dimensions of narrative engagement in narrative persuasion. *Communications*, 34, 385–405. doi:10.1515/COMM.2009.024

[CIT0014] De Graaf, A., Hoeken, H., Sanders, J., & Beentjes, J. W. J. (2012). Identification as a mechanism of narrative persuasion. *Communication Research*, 39, 802–823. doi:10.1177/0093650211408594

[CIT0015] Djikic, M., Oatley, K., Zoeterman, S., & Peterson, J. B. (2009). On being moved by art: How reading fiction transforms the self. *Creativity Research Journal*, 21, 24–29. doi:10.1080/10400410802633392

[CIT0016] Escalas, J. E. (2004). Imagine yourself in the product: Mental simulation, narrative transportation, and persuasion. *Journal of Advertising, 33*, 37–48.

[CIT0017] Faul, F., Erdfelder, E., Lang, A.-G., & Buchner, A. (2007). G*Power 3: A flexible statistical power analysis program for the social, behavioral, and biomedical sciences. *Behavior Research Methods*, 39, 175–191. 17695343 10.3758/bf03193146

[CIT0018] Gabriel, S., & Young, A. F. (2011). Becoming a vampire without being bitten: The narrative collective-assimilation hypothesis. *Psychological Science*, 22, 990–994. doi:10.1177/0956797611415541 21750250

[CIT0019] Gebbers, T., De Wit, J., & Appel, M. (2017). Transportation into narrative worlds and the motivation to change health-related behavior. *International Journal of Communication*, 11, 4886–4906.

[CIT0020] Gerrig, R. J. (1993). *Experiencing narrative worlds: On the psychological activities of reading*. New Haven, CT: Yale University Press.

[CIT0021] Gerrig, R. J., & Zimbardo, P. G. (2002). Self-concept. In R. J. Gerrig & P. G. Zimbardo (Eds.), *Glossary of psychological terms*. (Reprinted from *Psychology and life* (16th ed.) by R. J. Gerrig & P. G. Zimbardo, Eds., 2002, Boston, MA: Allyn & Bacon.) Retrieved from http://web.archive.org/web/20170924065421/http://www.apa.org/research/action/glossary.aspx?tab=18

[CIT0022] Green, M. C., & Brock, T. C. (2000). The role of transportation in the persuasiveness of public narratives. *Journal of Personality and Social Psychology*, 79, 701–721. 11079236 10.1037//0022-3514.79.5.701

[CIT0023] Green, M. C., & Brock, T. C. (2002). In the mind’s eye: Transportation-imagery model of narrative persuasion. In M. C. Green, J. J. Strange, & T. C. Brock (Eds.), *Narrative impact: Social and cognitive foundations* (pp. 315–341). Mahwah, NJ: Erlbaum.

[CIT0024] Green, M. C., Kass, S., Carrey, J., Herzig, B., Feeney, R. & Sabini, J. (2008). Transportation across media: Repeated exposure to print and film. *Media Psychology*, *11*, 512–539.

[CIT0025] Hartung, F., Burke, M., Hagoort, P., & Willems, R. M. (2016). Taking perspective: Personal pronouns affect experiential aspects of literary reading. *PLoS One*, 11, e0154732. doi:10.1371/journal.pone.0154732 27192060 PMC4883771

[CIT0026] Hayes, A. F. (2013). *Introduction to mediation, moderation, and conditional process analysis: A regression-based approach*. New York, NY: Guilford.

[CIT0027] Hayes, A. F. (2017). *The PROCESS macro for SPSS and SAS* [computer program]. Retrieved September 22, 2017, from http://processmacro.org/

[CIT0028] Horton, W. S., & Rapp, D. N. (2003). Out of sight, out of mind: Occlusion and the accessibility of information in narrative comprehension. *Psychonomic Bulletin & Review*, 10, 104–110. doi:10.3758/BF03196473 12747496

[CIT0029] Isaac, A. R., & Marks, D. F. (1994). Individual differences in mental imagery experience: Developmental changes and specialization. *British Journal of Psychology*, 85, 479–500. 7812670 10.1111/j.2044-8295.1994.tb02536.x

[CIT0030] Jerusalem, M., & Schwarzer, R. (1999). Allgemeine Selbstwirksamkeit [General Self-Efficacy]. In R. Schwarzer & M. Jerusalem (Eds.), *Skalen zur Erfassung von Lehrer- und Schülermerkmalen* (pp. 13–14). Berlin, Germany: Freie Universität Berlin.

[CIT0031] Kaufman, G. F., & Libby, L. K. (2012). Changing beliefs and behavior through experience-taking. *Journal of Personality and Social Psychology*, 103, 1–19. doi:10.1037/a0027525 22448888

[CIT0032] Krampen, G. (1979). Differenzierungen des Konstruktes der Kontrollüberzeugung. Deutsche Bearbeitung und Anwendung der IPC-Skalen. *Zeitschrift für Experimentelle und Angewandte Psychologie*, 26, 573–595.

[CIT0033] Levenson, H. (1974). Activism and powerful others: Distinctions within the concept of internal-external control. *Journal of Personality Assessment*, 38, 377–383. doi:10.1080/00223891.1974.10119988

[CIT0034] Lowry, R. (2018). The confidence interval of rho. Retrieved from http://vassarstats.net/rho.html

[CIT0035] Magliano, J. P., Taylor, H. A., & Kim, H. J. J. (2005). When goals collide: Monitoring the goals of multiple characters. *Memory & Cognition*, 33, 1357–1367. doi:10.3758/BF03193368 16615383

[CIT0036] Marks, D. F. (1973). Visual imagery differences in the recall of pictures. *British Journal of Psychology*, 64, 17–24. 4742442 10.1111/j.2044-8295.1973.tb01322.x

[CIT0037] Marks, D. F. (1999). Consciousness, mental imagery and action. *British Journal of Psychology*, 90, 567–585. doi:10.1348/000712699161639

[CIT0038] McKelvie, S. J. (1995). The VVIQ as a psychometric test of individual differences in visual imagery vividness: A critical quantitative review and plea for direction. *Journal of Mental Imagery*, 19, 1–106.

[CIT0039] McKelvie, S. J., & Demers, E. G. (1979). Individual differences in reported visual imagery and memory performance. *British Journal of Psychology*, 70, 51–57. 486866 10.1111/j.2044-8295.1979.tb02142.x

[CIT0040] Moyer‐Gusé, E. (2008). Toward a theory of entertainment persuasion: Explaining the persuasive effects of entertainment‐education messages. *Communication Theory*, 18, 407–425. doi:10.1111/comt.2008.18.issue-3

[CIT0041] Murphy, S. T., Frank, L. B., Chatterjee, J. S., & Baezconde‐Garbanati, L. (2013). Narrative versus nonnarrative: The role of identification, transportation, and emotion in reducing health disparities. *Journal of Communication*, 63, 116–137. doi:10.1111/jcom.2013.63.issue-1 PMC3857102 24347679

[CIT0042] Oatley, K. (1994). A taxonomy of the emotions of literary response and a theory of identification in fictional narrative. *Poetics*, 23, 53–74. doi:10.1016/0304-422X(94)P4296-S

[CIT0043] O’Brien, E. J., & Albrecht, J. E. (1992). Comprehension strategies in the development of a mental model. *Journal of Experimental Psychology: Learning, Memory, and Cognition*, 18, 777–784. 10.1037//0278-7393.18.4.777 1385615

[CIT0044] Paivio, A. (1971). *Imagery and verbal processes*. New York, NY: Holt, Rinehart &Winston.

[CIT0045] Paivio, A., & Harshman, R. (1983). Factor analysis of a questionnaire on imagery and verbal habits and skills. *Canadian Journal of Psychology*, 37, 461–483. doi:10.1037/h0080749

[CIT0046] Papa, M., Singhal, A., Law, S., Pant, S., Sood, S., Rogers, E. M., & Shefner-Rogers, C. L. (2000). Entertainment-education and social change: An analysis of parasocial interaction, social learning, collective efficacy, and paradoxical communication. *Journal of Communication*, 50, 31–55. doi:10.1111/j.1460-2466.2000.tb02862.x

[CIT0047] Paulus, C. (2009). Der Saarbrücker Persönlichkeitsfragebogen SPF (IRI) zur Messung von Empathie: Psychometrische Evaluation der deutschen Version des Interpersonal Reactivity Index. Retrieved from http://hdl.handle.net/20.500.11780/3343

[CIT0048] Richter, T., Appel, M., & Calio, F. (2014). Stories can influence the self-concept. *Social Influence*, 9, 172–188. doi:10.1080/15534510.2013.799099

[CIT0049] Rogers, E. M., Vaughan, P. W., Swalehe, R. M. A., Rao, N., Svenkerud, P., & Sood, S. (1999). Effects of an entertainment-education radio soap opera on family planning in Tanzania. *Studies in Family Planning*. 30, 193–211. 10546311 10.1111/j.1728-4465.1999.00193.x

[CIT0050] Rotter, J. B. (1966). Generalized expectancies for internal versus external control of reinforcement. *Psychological Monographs: General and Applied*, 80, 1–28. 5340840

[CIT0051] Sabido, M. (1981). Towards the social use of soap operas. Mexico City, Mexico: Institute for Communication Research.

[CIT0052] Sestir, M., & Green, M. C. (2010). You are who you watch: Identification and transportation effects on temporary self-concept. *Social Influence*, 5, 272–288. doi:10.1080/15534510.2010.490672

[CIT0053] Shaughnessy, J. J., Zechmeister, E. B., & Zechmeister, J. (2003). *Research methods in psychology* (6th ed.). New York, NY: McGraw Hill.

[CIT0054] Shedlosky-Shoemaker, R., Costabile, K. A., DeLuca, H. K., & Arkin, R. M. (2011). The social experience of entertainment media. *Journal of Media Psychology: Theories, Methods, and Applications*, 23, 111–121. doi:10.1027/1864-1105/a000042

[CIT0055] Singhal, A., & Rogers, E. M. (1999). *Entertainment-education: A communication strategy for social change*. Mahwah, NJ: Lawrence Erlbaum Associates.

[CIT0056] Slater, M. D., & Rouner, D. (2002). Entertainment-education and elaboration-likelihood: Understanding the processing of narrative persuasion. *Communication Theory*, 12, 173–191.

[CIT0057] SPSS Tutorials. (2018). SPSS confidence intervals for correlations tool. Retrieved from https://www.spss-tutorials.com/spss-confidence-intervals-for-correlations-tool/

[CIT0058] Sukalla, F., Bilandzic, H., Bolls, P. D., & Busselle, R. W. (2016). Embodiment of narrative engagement: Connecting self-reported narrative engagement to psychophysiological measures. *Journal of Media Psychology*, 28, 175–186. doi:10.1027/1864-1105/a000153

[CIT0059] Tal-Or, N., & Cohen, J. (2016). Unpacking engagement: Convergence and divergence in transportation and identification. *Annals of the International Communication Association*, 40, 33–66. doi:10.1080/23808985.2015.11735255

[CIT0060] Tsai, J. L., Louie, J. Y., Chen, E. E., & Uchida, Y. (2007). Learning what feelings to desire: Socialization of ideal affect through children’s storybooks. *Personality and Social Psychology Bulletin*, 33, 17–30. doi:10.1177/0146167206292749 17178927

[CIT0061] Tukachinsky, R. (2014). Experimental manipulation of psychological involvement with media. *Communication Methods and Measures*, 8, 1–33. doi:10.1080/19312458.2013.873777

[CIT0062] Uanhoro, J. (2018). Effect size calculators. Retrieved from https://effect-size-calculator.herokuapp.com/$partial-eta-squared-fixed-effects

[CIT0063] Vaughan, P. W., Rogers, E. M., Singhal, A., & Swalehe, R. M. (2000). Entertainment-education and HIV/AIDS prevention: A field experiment in Tanzania. *Journal of Health Communication*, 5, 81–100. doi:10.1080/10810730050019573 11010359

[CIT0064] Wang, J., & Calder, B. J. (2006). Media transportation and advertising. *Journal of Consumer Research*, 33, 151–162. doi:10.1086/506296

[CIT0065] Wuensch, K. (2009). Confidence intervals for squared effect size estimates in ANOVA: What confidence coefficient should be employed? Retrieved from http://core.ecu.edu/psyc/wuenschk/docs30/CI-Eta2-Alpha.doc

[CIT0066] Zwarun, L., & Hall, A. (2012). Narrative persuasion, transportation, and the role of need for cognition in online viewing of fantastical films. *Media Psychology*, 15, 327–355. doi:10.1080/15213269.2012.700592

